# The 50 most-cited publications in lymphatic surgery: A bibliometric analysis

**DOI:** 10.1016/j.jpra.2026.03.032

**Published:** 2026-03-28

**Authors:** Abdullah A. Al Qurashi, Pharel Njessi, Hatan Mortada, Olivier Camuzard, Joon Pio Hong, Elise Lupon

**Affiliations:** a Department of Plastic and Reconstructive Surgery, Institut Universitaire Locomoteur et du Sport, University Côte d’Azur, Nice, France; b Université Côte d’Azur, CNRS, LP2M, Nice, France; c Ministry of Health, Mecca Health Cluster, Mecca, Saudi Arabia; d Department of Plastic Surgery & Burn Unit, King Saud Medical City, Riyadh, Saudi Arabia; e Division of Plastic Surgery, Department of Surgery, King Saud University Medical City, King Saud University, Riyadh, Saudi Arabia; f Department of Plastic and Reconstructive Surgery, University of Ulsan College of Medicine, Asan Medical Center, Seoul, Korea

**Keywords:** Lymphatic surgery, Lymphedema, Bibliometric analysis, Lymphaticovenous anastomosis, Vascularized lymph node transfer, Supermicrosurgery

## Abstract

**Background:**

Lymphatic surgery has evolved from palliative debulking procedures to physiologic reconstruction using microsurgical and supermicrosurgical techniques. Despite this progress, the publications that have most influenced this evolution have not been systematically examined. This study aimed to analyze the 50 most-cited publications in lymphatic surgery, assessing their bibliometric characteristics, study designs, levels of evidence, and thematic contributions to lymphedema surgical management.

**Methods:**

The Web of Science Core Collection was searched for articles on surgical treatment of lymphedema from inception to November 7, 2025. The 50 most-cited articles were identified and analyzed for citation count, citation density (citations per year), authorship, geographic origin, journal distribution, and keyword frequency. Studies were categorized by design and evaluated according to the Oxford Centre for Evidence-Based Medicine levels of evidence. Concise summaries were generated to facilitate access to key findings.

**Results:**

Published between 2004 and 2023, the top 50 articles accumulated 4612 citations (mean 92.2, median 75.5). Citation density ranged from 3.9 to 32.5 citations per year. Plastic and Reconstructive Surgery was the most represented journal (*n* = 14). Asian institutions predominated, with Japan (*n* = 16) and Taiwan (*n* = 11) accounting for 54% of publications. Case series were the most common study design (*n* = 24). Most studies provided Level 4 or 5 evidence (74%), although two Level 2 randomized controlled trials were identified. Keyword analysis identified supermicrosurgery, lymphaticovenular anastomosis, and indocyanine green lymphography as the most recurring technical concepts.

**Conclusions:**

This bibliometric analysis highlights seminal publications underlying the development of supermicrosurgical techniques such as lymphaticovenous anastomosis and vascularized lymph node transfer. The results demonstrate a strong Eastern contribution to innovation. While observational studies predominate, the near-total absence of Level 1 evidence and the presence of only two Level 2 trials underscore the need for well-designed prospective comparative studies to establish standardized treatment algorithms.

Level of evidence: IV

## Introduction

Lymphedema is a chronic, progressive, and debilitating condition resulting from the failure of the lymphatic transport system, leading to the accumulation of protein-rich interstitial fluid.^1^ Clinically characterized by limb swelling, chronic inflammation, fibrosis, and recurrent cellulitis, the disease imposes a substantial burden on patients’ quality of life, affecting physical function and psychological well-being.^2^^,^^3^

The global prevalence is significant, particularly in the context of cancer survivorship, with breast cancer-related lymphedema affecting approximately one in five survivors.^4^ Historically, management was limited to conservative measures such as Complex Decongestive Therapy (CDT) or reductive surgeries with high morbidity.^5^ However, the past 2 decades have witnessed a paradigm shift with microsurgical and supermicrosurgical techniques, moving the field toward physiologic reconstruction.^6^ Lymphaticovenous anastomosis (LVA) and vascularized lymph node transfer (VLNT) have become the cornerstones of this modern approach. LVA, a supermicrosurgical technique that bypasses the obstruction by shunting lymph into the venous system, has shown efficacy in early-stage fluid-predominant disease.^7^^,^^8^ Conversely, VLNT, which involves the transfer of healthy lymph nodes to the affected limb, is often indicated for more advanced stages and relies on lymphangiogenesis and internal pumping mechanisms.^9^^,^^10^ These physiologic procedures are frequently integrated with suction-assisted lipectomy (liposuction) to address the irreversible fibro-adipose deposition found in chronic stages.^11^

Despite this rapid clinical evolution, the scientific literature remains fragmented across plastic surgery, oncology, and vascular journals. Bibliometric analysis provides a quantitative method to evaluate scientific impact, identifying the most influential articles that have shaped current understanding.^12^ The objective of this study was to identify the 50 most-cited articles on lymphatic surgery, including LVA, VLNT, and debulking procedures. Furthermore, we aim to provide a concise summary of each included study to facilitate quick access to key findings for clinicians and researchers.

## Methodology

### Study selection and classification

Inclusion criteria were defined as original articles or reviews published in English involving human subjects. Although our institutional context is francophone, no French-language articles met the citation threshold for inclusion in the final top 50, and the analysis was therefore conducted exclusively on English-language publications. The study scope was limited to surgical interventions for lymphedema, specifically including LVA, VLNT, lymphatic vessel transfer, suction-assisted lipectomy, and Charles procedures. Exclusion criteria encompassed studies focusing exclusively on sentinel lymph node biopsy, oncologic staging, or diagnostic mapping. Furthermore, records describing purely non-surgical management (e.g., complex decongestive therapy), animal models, or basic science research without direct clinical application were excluded. Editorials and letters were also excluded (Supplementary material 1). Following selection, the articles were categorized by study design into case series, reviews, retrospective observational studies, case reports, prospective observational studies, randomized controlled trials, consensus guidelines, and technical papers. The sample size was recorded for each study where applicable. Clinical articles were evaluated using the Oxford Centre for Evidence-Based Medicine (OCEBM) level of evidence system.^13^

### Search strategy

**Figure 1:**
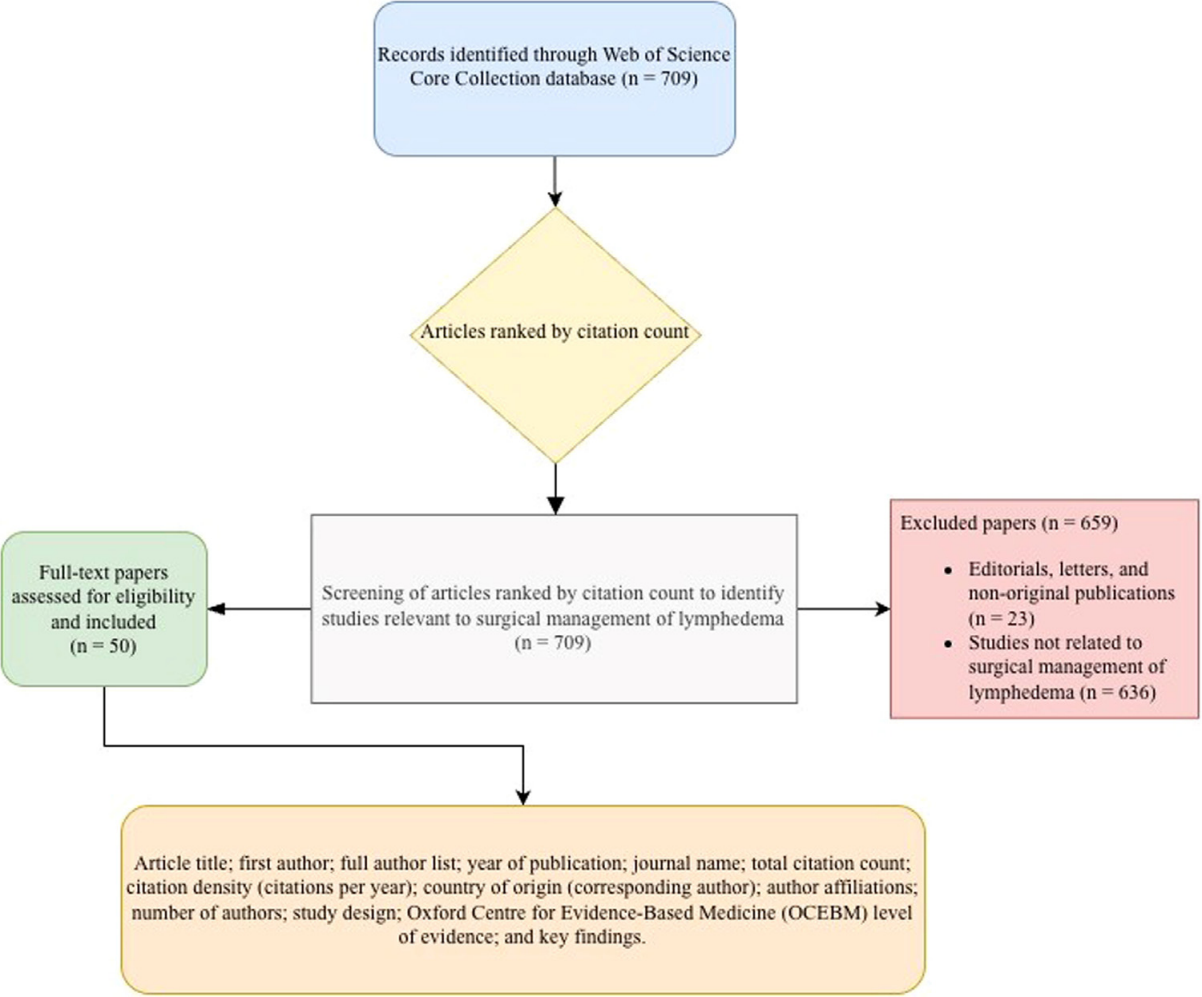
Flowchart of the methodology of creation of the database of the 50 most-cited lymphatic surgery articles.

The 50 most-cited articles relating to lymphatic surgery were extracted from the Web of Science Core Collection database on November 7, 2025. The following search string was used: TS = (“lymphatic surgery” OR “lymphatic reconstruction” OR “lymphovenous anastomosis” OR “lymphaticovenous anastomosis” OR “lymphovenous bypass” OR “lymphaticovenous bypass” OR “vascularized lymph node transfer” OR “lymph node transfer” OR “lymphatic vessel transfer” OR “lymphatic flap” OR “supermicrosurgery” OR “suction-assisted lipectomy” OR “liposuction” OR “Charles procedure” OR “modified Charles procedure” OR “debulking surgery” OR “lymphatic drainage restoration”) AND TS = (“lymphedema” OR “lymphoedema”). To ensure specificity, the following exclusion terms were applied using the NOT operator: NOT TS = (“sentinel lymph node biopsy” OR “oncologic staging” OR “manual lymph drainage” OR “complex decongestive therapy” OR “pneumatic compression”) (Supplementary material 1). The initial search yielded 709 records. Following filtering by document type and language, the results were sorted by total citation count. A multi-step screening process was conducted: 23 records were removed initially for being outside the scope or lacking original data (e.g., editorials), and 73 records were screened in detail to identify the final 50 most-cited articles (Figure 1).

### Data collection and analysis

Data were collected and analyzed on a computerized spreadsheet (Microsoft Excel, Microsoft Corporation, Redmond, WA). For each of the included reports, the following data were recorded: title; full authors’ names; year of publication; source journal; country of origin; sample size; and total number of citations. The country of origin refers to the affiliation of the corresponding author. Additional derived data included citation density (citations per year, calculated as total citations divided by years since publication through 2025), institutional affiliation, and key findings. A keyword analysis was performed using Web of Science–indexed Author Keywords and Keywords Plus to identify recurring thematic concepts across the 50 articles.

## Results

### Citation profile and journal distribution

**Figure 2:**
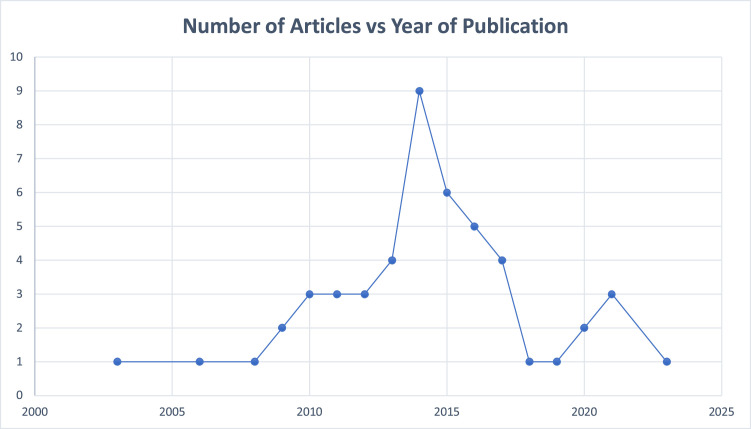
The number of articles published over the years among the 50 most-cited lymphatic surgery articles.

**Figure 3:**
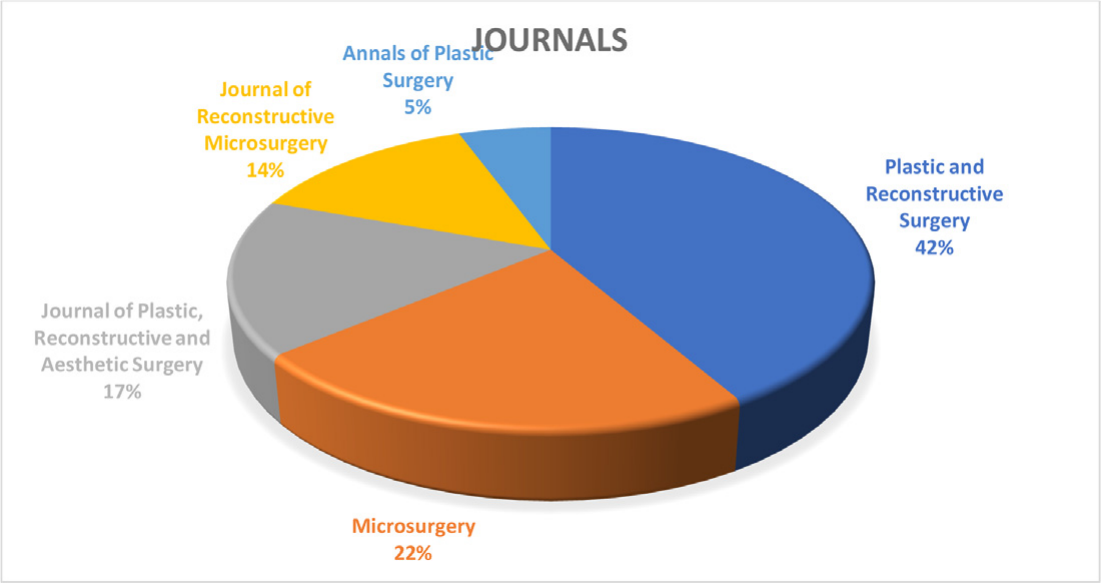
The five journals that contributed largest number of publications to the 50 most-cited lymphatic surgery articles.

The 50 most-cited articles in lymphatic surgery accumulated a total of 4612 citations; the citation counts varied across the articles, with a mean of 92.2 and a median of 75.5 citations per article. Citation density ranged from 3.9 to 32.5 citations per year (Coriddi et al.^14^), with a mean of 9.8 and a median of 7.7 citations per year. Notably, when normalized for time, the highest-impact articles differed from those with the highest absolute citation counts: the randomized trial by Coriddi et al. (32.5 citations/year), the robotic surgery trial by van Mulken et al. (23.8 citations/year), and the comprehensive review by Schaverien and Coroneos (23.3 citations/year) emerged as the most rapidly cited publications, highlighting the current relevance of immediate lymphatic reconstruction and technological innovation. Articles were published between 2004 and 2023. Annual output increased gradually through the late 2000s and early 2010s, followed by a peak in 2014 (*n* = 10). Publication volume remained relatively high in 2015–2016 (*n* = 6 and *n* = 6, respectively), then declined to low single-digit counts per year thereafter (Figure 2). Plastic and Reconstructive Surgery published the highest number of articles (*n* = 14), followed by Microsurgery (*n* = 8), Annals of Plastic Surgery (*n* = 5), Journal of Plastic, Reconstructive & Aesthetic Surgery (*n* = 5), and the Journal of Reconstructive Microsurgery (*n* = 4) (Figure 3).

### Geographic and institutional distribution

**Figure 4:**
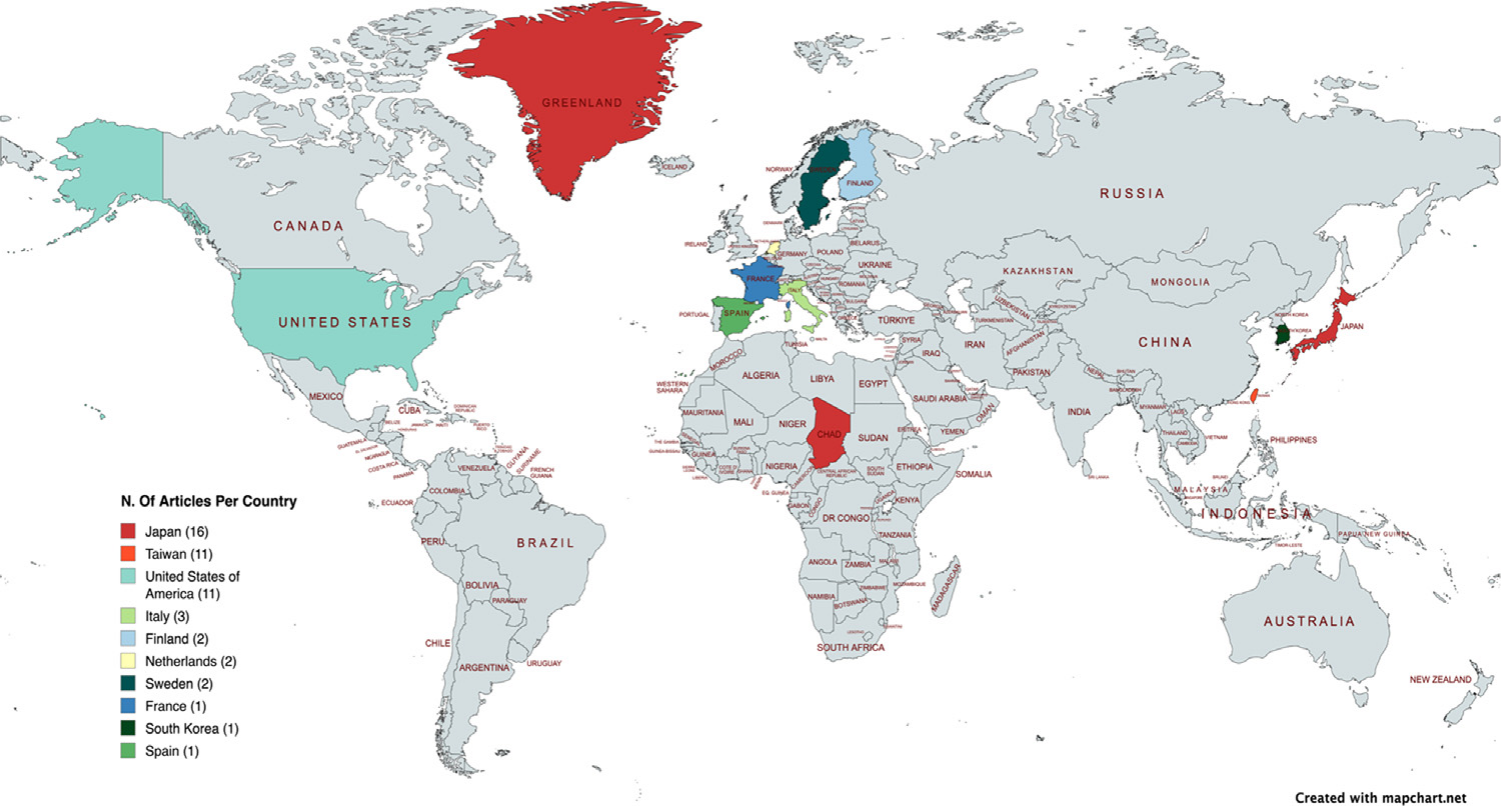
The geographical distribution of the origins of the 50 most-cited lymphatic surgery articles.

**Figure 5:**
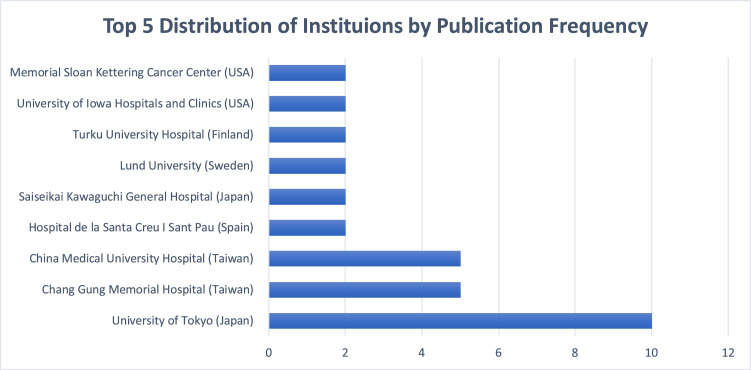
Top 5 institutions by publication frequency among the 50 most-cited lymphatic surgery articles for lymphedema.

The included articles originated from 10 countries. Japan led with 16 publications, followed by Taiwan (*n* = 11) and the United States (*n* = 11) (Figure 4). The University of Tokyo was the most prolific institution (*n* = 10), followed by Chang Gung Memorial Hospital (*n* = 5) and China Medical University Hospital (*n* = 5). Memorial Sloan Kettering Cancer Center, Lund University, and Turku University Hospital each contributed 2 articles (Figure 5).

### Keyword and thematic analysis

Keyword analysis was performed using Author Keywords (available for 24 of 50 articles) and Web of Science–generated Keywords Plus (available for 48 of 50) (Table 1). The most frequent Author Keywords, excluding the generic term “lymphedema,” were “supermicrosurgery” (*n* = 11), “lymphaticovenular anastomosis” (*n* = 7), “breast cancer” (*n* = 4), “indocyanine green” (*n* = 3), and “liposuction” (*n* = 3). Keywords Plus analysis revealed that “lower-extremity lymphedema” (*n* = 14), “postmastectomy lymphedema” (*n* = 11), “lymphaticovenous anastomosis” (*n* = 8), “node transfer” (*n* = 8), and “indocyanine green lymphography” (*n* = 7) were the dominant indexed terms. These data confirm that supermicrosurgical techniques and ICG-guided approaches represent the central thematic pillars of the most-cited lymphatic surgery literature.

**Table 1:** Most frequent author keywords and keywords plus terms among the 50 most-cited articles in lymphatic surgery.

Rank	Keyword	Frequency (*n*)	Source
*Author keywords (available for 24 of 50 articles, excluding “lymphedema”)*			
1	Supermicrosurgery	11	Author keyword
2	Lymphaticovenular anastomosis	7	Author keyword
3	Breast cancer	4	Author keyword
4	Indocyanine green	3	Author keyword
4	Liposuction	3	Author keyword
*Keywords Plus (available for 48 of 50 articles)*			
1	Lower-extremity lymphedema	14	Keywords plus
2	Postmastectomy lymphedema	11	Keywords plus
3	Lymphaticovenous anastomosis	8	Keywords plus
3	Node transfer	8	Keywords plus
5	Indocyanine green lymphography	7	Keywords plus

Author Keywords are designated by the authors of each article. Keywords Plus are terms automatically generated by Web of Science based on the titles of cited references. The generic term “lymphedema” was excluded from the Author Keywords ranking.

### Study design and level of evidence

The number of authors listed per manuscript ranged from 2 to 12 (mean 6.24, median 6). Based on the Oxford Centre for Evidence-Based Medicine (OCEBM) level of evidence scale, the majority of articles were classified as Level 4 evidence (*n* = 24; 48%), followed by Level 5 (*n* = 13; 26%) and Level 3 (*n* = 11; 22%). Two articles (4%) were classified as Level 2, and no Level 1 studies were identified (Table 2). Regarding study design, case series were the most frequent publication type (*n* = 24), followed by non-randomized comparative studies (*n* = 11), narrative reviews and consensus documents (*n* = 9), case reports (*n* = 4), and randomized controlled trials (*n* = 2) (Table 3). Details on the study design, sample size, and characteristics of each included article are provided in Table 4, with an accompanying study summary in the supplementary material (Supplementary Material 2).

**Table 2:** OCEBM levels of evidence among the 50 most-cited lymphatic surgery articles.

OCEBM level	Number of articles	Percent
Level 1	0	0.0%
Level 2	2	4.0%
Level 3	11	22.0%
Level 4	24	48.0%
Level 5	13	26.0%
Total	**50**	**100.0%**

**Table 3:** Study design distribution of the 50 most-cited lymphatic surgery articles.

Rank	Study design	Number of articles
1	Case series	24
2	Non-randomized comparative studies	11
3	Narrative reviews and consensus documents	9
4	Case report	4
5	Randomized controlled trial	2

**Table 4:** Study characteristics of the 50 most-cited lymphatic surgery articles, ranked by total citations (WoS) (*n* = 50).

Rank	Title	First author	Year	Journal	Country/Region	Total citations	Study design/Publication type	OCEBM level	Sample size (*n*)
1	Vascularized Groin Lymph Node Transfer Using the Wrist as a Recipient Site for Management of Postmastectomy Upper Extremity Lymphedema	Lin CH	2009	Plastic and Reconstructive Surgery	Taiwan	290	Case Series (Retrospective)	Level 4	13
2	Vascularized Groin Lymph Node Flap Transfer for Postmastectomy Upper Limb Lymphedema: Flap Anatomy, Recipient Sites, and Outcomes	Cheng MH	2013	Plastic and Reconstructive Surgery	Taiwan	246	Non-randomized Controlled Trial (Comparative Cohort)	Level 3	10 + 10 cadavers + 10 controls
3	Lymphedema: Surgical and Medical Therapy	Chang DW	2016	Plastic and Reconstructive Surgery	USA	155	Narrative Review/Expert Opinion	Level 5	N/A
4	Complications of Autologous Lymph-node Transplantation for Limb Lymphoedema	Vignes S	2013	European Journal of Vascular and Endovascular Surgery	France	149	Case Series (Prospective)	Level 4	26 (34 transplants)
5	Surgical Treatment of Lymphedema	Schaverien MV	2019	Plastic and Reconstructive Surgery	USA	140	Narrative Review	Level 5	N/A
6	Microlymphatic Surgery for the Treatment of Iatrogenic Lymphedema	Becker C	2012	Clinics in Plastic Surgery	USA	140	Narrative Review/Technique Description	Level 5	N/A
7	Perforator Flaps and Supermicrosurgery	Koshima I	2010	Clinics in Plastic Surgery	Japan	137	Narrative Review/Technique Description	Level 5	N/A
8	Minimal invasive lymphaticovenular anastomosis under local anesthesia for leg lymphedema	Koshima I	2004	Annals of Plastic Surgery	Japan	137	Case Series	Level 4	52
9	Minimally Invasive Lymphatic Supermicrosurgery (MILS): Indocyanine Green Lymphography-Guided Simultaneous Multisite Lymphaticovenular Anastomoses via Millimeter Skin Incisions	Yamamoto T	2014	Annals of Plastic Surgery	Japan	129	Case Series	Level 4	11 limbs
10	First-in-human robotic supermicrosurgery using a dedicated microsurgical robot for treating breast cancer-related lymphedema: a randomized pilot trial	van Mulken TJM	2020	Nature Communications	Netherlands	119	Randomized Controlled Trial (Pilot)	Level 2	20 (8 robot + 12 manual)
11	A case of donor-site lymphoedema after lymph node superficial circumflex iliac artery perforator flap transfer	Pons G	2014	Journal of Plastic, Reconstructive & Aesthetic Surgery	Italy	116	Case Report	Level 5	1
12	Donor-Site Lymphatic Function after Microvascular Lymph Node Transfer	Viitanen TP	2012	Plastic and Reconstructive Surgery	Finland	110	Case Series (Prospective)	Level 4	13
13	Vascularized Lymph Node Transfer Based on the Hilar Perforators Improves the Outcome in Upper Limb Lymphedema	Bassiri Gharb B	2011	Annals of Plastic Surgery	Taiwan	101	Non-randomized Comparative Study (Retrospective Cohort)	Level 3	21
14	The Intravascular Stenting Method for Treatment of Extremity Lymphedema with Multiconfiguration Lymphaticovenous Anastomoses	Narushima M	2010	Plastic and Reconstructive Surgery	Japan	100	Case Series	Level 4	14 (39 anastomoses)
15	Multisite Lymphaticovenular Bypass Using Supermicrosurgery Technique for Lymphedema Management in Lower Lymphedema Cases	Mihara M	2016	Plastic and Reconstructive Surgery	Japan	94	Case Series (Retrospective)	Level 4	84 (162 limbs)
16	Microsurgery for treatment of peripheral lymphedema: Long-term outcome and future perspectives	Campisi C	2007	Microsurgery	Italy	93	Case Series (Retrospective)	Level 4	>1500
17	Current Concepts in the Surgical Management of Lymphedema	Kung TA	2017	Plastic and Reconstructive Surgery	USA	91	Narrative Review (CME Article)	Level 5	N/A
18	Barcelona Consensus on Supermicrosurgery	Masia J	2014	Journal of Reconstructive Microsurgery	Spain	89	Consensus/Guideline	Level 5	N/A
19	Comparison of Vascularized Supraclavicular Lymph Node Transfer and Lymphaticovenular Anastomosis	Akita S	2015	Annals of Plastic Surgery	Japan	89	Non-randomized Comparative Study (Retrospective Cohort)	Level 3	46
20	Quality of life following liposuction and conservative treatment of arm lymphedema	Brorson H	2006	Lymphology	Sweden	89	Non-randomized Comparative Study (Prospective Cohort)	Level 3	49 (35 lipo + 14 CCT)
21	From Theory to Evidence: Long-Term Evaluation of the Mechanism of Action and Flap Integration of Distal Vascularized Lymph Node Transfers	Patel KM	2015	Journal of Reconstructive Microsurgery	Taiwan	89	Case Series (Retrospective analysis of prospectively collected data)	Level 4	20
22	Simultaneous multi-site lymphaticovenular anastomoses for primary lower extremity and genital lymphoedema complicated with severe lymphorrhea	Yamamoto T	2011	Journal of Plastic, Reconstructive & Aesthetic Surgery	Japan	89	Case Report (*n* = 2)	Level 5	2
23	Lymphaticovenous anastomosis to prevent cellulitis associated with lymphoedema	Mihara M	2014	British Journal of Surgery	Japan	86	Case Series (Retrospective)	Level 4	95
24	A modified side-to-end lymphaticovenular anastomosis	Yamamoto T	2013	Microsurgery	Japan	78	Case Series	Level 4	14 limbs
25	Lymph Flow Restoration after Tissue Replantation and Transfer: Importance of Lymph Axiality and Possibility of Lymph Flow Reconstruction without Lymph Node Transfer or Lymphatic Anastomosis	Yamamoto T	2018	Plastic and Reconstructive Surgery	Japan	76	Case Series (Retrospective)	Level 4	38
26	Near-infrared illumination system-integrated microscope for supermicrosurgical lymphaticovenular anastomosis	Yamamoto T	2014	Microsurgery	Japan	75	Non-randomized Comparative Study (Cohort)	Level 3	12 (40 LVAs)
27	Overview of Lymph Node Transfer for Lymphedema Treatment	Ito R	2014	Plastic and Reconstructive Surgery	USA	74	Narrative Review	Level 5	N/A
28	Outcomes of Lymphedema Microsurgery for Breast Cancer-related Lymphedema With or Without Microvascular Breast Reconstruction	Engel H	2018	Annals of Surgery	Taiwan	74	Non-randomized Comparative Study (Retrospective Cohort)	Level 3	124
29	Modified Charles procedure and lymph node flap transfer for advanced lower extremity lymphedema	Sapountzis S	2014	Microsurgery	Taiwan	73	Case Series	Level 4	24
30	Changing the Paradigm: Lymphovenous Anastomosis in Advanced Stage Lower Extremity Lymphedema	Cha HG	2021	Plastic and Reconstructive Surgery	South Korea	72	Case Series (Retrospective)	Level 4	42 (50 limbs)
31	Circumferential suction-assisted lipectomy for lymphoedema after surgery for breast cancer	Damstra RJ	2009	British Journal of Surgery	Netherlands	71	Case Series (Prospective)	Level 4	37
32	Evaluating the Impact of Immediate Lymphatic Reconstruction for the Surgical Prevention of Lymphedema	Johnson AR	2021	Plastic and Reconstructive Surgery	USA	70	Case Series (Retrospective)	Level 4	97 (32 ILR)
33	Complete lymph flow reconstruction: A free vascularized lymph node true perforator flap transfer with efferent lymphaticolymphatic anastomosis	Yamamoto T	2016	Journal of Plastic, Reconstructive & Aesthetic Surgery	Japan	68	Case Report (*n* = 1)	Level 5	1
34	Controlled compression and liposuction treatment for lower extremity lymphedema	Brorson H	2008	Lymphology	Sweden	66	Case Report (*n* = 1)	Level 5	N/R
35	Surgical management of lymphedema: past, present, and future	Mehrara BJ	2011	Lymphatic Research and Biology	USA	66	Narrative Review	Level 5	N/A
36	Navigation lymphatic supermicrosurgery for iatrogenic lymphorrhea: Supermicrosurgical lymphaticolymphatic anastomosis and lymphaticovenular anastomosis under indocyanine green lymphography navigation	Yamamoto T	2014	Journal of Plastic, Reconstructive & Aesthetic Surgery	Japan	66	Non-randomized Comparative Study (Retrospective)	Level 3	4
37	Efficacy of Immediate Lymphatic Reconstruction to Decrease Incidence of Breast Cancer-related Lymphedema	Coriddi M	2023	Annals of Surgery	USA	65	Randomized Controlled Trial (RCT)	Level 2	144 (72 + 72)
38	The lymphatic superficial circumflex iliac vessels deep branch perforator flap: A new preventive approach to lower limb lymphedema after groin dissection-preliminary evidence	Gentileschi S	2017	Microsurgery	Italy	62	Prospective Controlled Study (Split-body design)	Level 3	5
39	Navigation Lymphatic Supermicrosurgery for the Treatment of Cancer-Related Peripheral Lymphedema	Yamamoto T	2014	Vascular and Endovascular Surgery	Japan	60	Case Series	Level 4	8 (21 LVAs)
40	Indication of Lymphaticovenous Anastomosis for Lower Limb Primary Lymphedema	Hara H	2015	Plastic and Reconstructive Surgery	Japan	59	Case Series (Retrospective)	Level 4	62 (79 limbs)
41	Efferent Lymphatic Vessel Anastomosis: Supermicrosurgical Efferent Lymphatic Vessel-to-Venous Anastomosis for the Prophylactic Treatment of Subclinical Lymphedema	Yamamoto T	2016	Annals of Plastic Surgery	Japan	59	Case Series	Level 4	14 legs
42	Successful treatment of early-stage lower extremity lymphedema with side-to-end lymphovenous anastomosis with indocyanine green lymphography assisted	Ito R	2016	Microsurgery	Taiwan	59	Case Series	Level 4	5
43	Indocyanine Green Lymphographic Evidence of Surgical Efficacy Following Microsurgical and Supermicrosurgical Lymphedema Reconstructions	Chen WF	2016	Journal of Reconstructive Microsurgery	USA	58	Case Series (Longitudinal)	Level 4	21
44	Double gastroepiploic vascularized lymph node transfers to middle and distal limb for the treatment of lymphedema	Ciudad P	2017	Microsurgery	Taiwan	58	Case Series (*n* = 7)	Level 4	7
45	Free lymph node flap transfer and laser-assisted liposuction: a combined technique for the treatment of moderate upper limb lymphedema	Nicoli F	2015	Lasers in Medical Science	Taiwan	56	Case Series	Level 4	10
46	Comparison of Outcomes between Side-to-End and End-to-End Lymphovenous Anastomoses for Early-Grade Extremity Lymphedema	AlJindan FK	2019	Plastic and Reconstructive Surgery	Taiwan	55	Non-randomized Comparative Study (Retrospective Cohort)	Level 3	58
47	Risk of donor-site lymphatic vessel dysfunction after microvascular lymph node transfer	Sulo E	2015	Journal of Plastic, Reconstructive & Aesthetic Surgery	Finland	55	Non-randomized Comparative Study (Historical Cohort)	Level 3	29 (13 + 16)
48	Lymphedema	Maclellan RA	2014	Seminars in Pediatric Surgery	USA	54	Narrative Review	Level 5	N/A
49	The “Octopus” Lymphaticovenular Anastomosis: Evolving Beyond the Standard Supermicrosurgical Technique	Chen WF	2015	Journal of Reconstructive Microsurgery	USA	53	Case Series (Pilot)	Level 4	9
50	A prospective study on combined lymphedema surgery: Gastroepiploic vascularized lymph nodes transfer and lymphaticovenous anastomosis followed by suction lipectomy	Di Taranto G	2021	Microsurgery	Taiwan	52	Prospective Non-randomized Comparative Study	Level 3	37 (21 + 16)

Country denotes the affiliation of the corresponding author. OCEBM, Oxford Centre for Evidence-Based Medicine; LVA, lymphaticovenous anastomosis; VLNT, vascularized lymph node transfer; RCT, randomized controlled trial; CCT, controlled compression therapy; ILR, immediate lymphatic reconstruction; N/A, not applicable (review/consensus articles); N/R, not reported.

## Discussion

The present study identified the 50 most influential articles in lymphatic surgery, representing a cumulative total of 4612 citations. The substantial citation count underscores the rapid expansion of this field within plastic and reconstructive surgery. Plastic and Reconstructive Surgery published the highest number of articles (28%), a finding consistent with other bibliometric analyses in the specialty,^15^ confirming its role as the primary journal for mainstreaming lymphedema microsurgery. The significant contribution of Microsurgery (*n* = 8) and the Journal of Reconstructive Microsurgery (*n* = 4) warrants specific acknowledgment; these specialty journals serve as primary outlets for detailed operative descriptions and highly technical reports that may achieve high citation counts within a relatively focused readership, reflecting the technical complexity inherent to supermicrosurgical procedures.^7^

### Geographic distribution and centers of excellence

A defining characteristic of this bibliometric study is the significant geographic predominance of Asian nations. Japan (*n* = 16) and Taiwan (*n* = 11) collectively contributed 54% of the most-cited articles. This “Eastern dominance” is largely driven by the focused publications of key centers of excellence. The University of Tokyo has been central to the advancement of supermicrosurgical lymphaticovenous anastomosis (LVA),^7^^,^^16^ while Chang Gung Memorial Hospital and China Medical University Hospital have played critical roles in refining vascularized lymph node transfer (VLNT), notably by introducing novel techniques such as the submental lymph node flap and the gastroepiploic node flap.^17^^,^^18^ This contrasts with the US and Europe, where earlier contributions focused more heavily on debulking and conservative therapy,^11^ though Western centers have recently led the shift toward immediate lymphatic reconstruction.^19^

### Analysis of the most influential articles

The five most-cited articles gathered a combined total of 980 citations, highlighting central themes such as^1^ distal lymph node transfer,^2^ stage-based treatment algorithms, and^3^ surgical safety. The most cited publication, a landmark case series by Lin et al.^20^ (290 citations), introduced the concept of “distal” VLNT by transferring a groin flap to the wrist. In this publication, the authors proposed the “pump mechanism” hypothesis and demonstrated significant limb reduction and decreased cellulitis, setting a benchmark for physiologic outcomes.^9^ Following closely, Cheng et al.^18^ (246 citations) established the anatomical foundation for the groin lymph node flap. This study is widely cited for defining the vascular preservation required, specifically the deep branch of the superficial circumflex iliac artery, to ensure node survival and donor safety. Providing a broader clinical framework, Chang et al.^6^ (155 citations) categorized surgical options into physiologic versus reductive procedures and proposed stage-based treatment algorithms consistent with international consensus.^1^ Vignes et al.^21^ (149 citations) contributed a pivotal safety study reporting the risk of iatrogenic lymphedema in the donor limb following VLNT, a finding that prompted the global adoption of reverse lymphatic mapping.^22^ Finally, a comprehensive review by Schaverien and Coroneos^23^ (140 citations) provided a detailed synthesis of surgical techniques, including lymphovenous bypass, VLNT, and liposuction, consolidating the evidence base for modern lymphedema surgery. Notably, the technical basis of supermicrosurgery was established by Koshima et al.^16^ (137 citations), whose comprehensive review formally defined “supermicrosurgery” as the anastomosis of vessels smaller than 0.8 mm; although this article ranked seventh, its conceptual influence on the field remains foundational. It is worth noting, however, that the field has increasingly shifted away from groin-based VLNT due to concerns over donor-site lymphedema risk and seroma rates.^21^^,^^24^ Contemporary practice has moved toward alternative donor sites and SCIP-based lymphatic flap approaches that incorporate lymphatic vessel transfer, such as the lymphatic superficial circumflex iliac artery perforator (L-SCIP) flap described by Gentileschi et al.,^25^ as well as combined approaches integrating VLNT with LVA and suction lipectomy.^26^ This evolution illustrates a key limitation of citation-based analyses, whereby historically influential techniques may no longer fully reflect current clinical practice.

### Evolution of techniques and levels of evidence

The chronological peak in 2014 should be interpreted with caution: while it is evident within the subset of highly cited articles and coincides with the widespread adoption of indocyanine green (ICG) lymphography, it does not necessarily reflect the overall trajectory of lymphatic surgery research, which has expanded dramatically in recent years.^27^ Our keyword analysis substantiates the centrality of ICG-guided techniques, with “indocyanine green lymphography” ranking among the most frequent Keywords Plus terms (*n* = 7) across the 50 articles; ICG revolutionized the field by allowing real-time visualization of lymphatic channels and lowering the technical barrier for LVA.^27^ Despite the major focus on physiologic reconstruction, the persistence of highly cited works on liposuction confirms that reductive techniques remain essential for late-stage, non-pitting disease where fibrosis predominates.^11^ Furthermore, the list captures the emerging option of “Immediate Lymphatic Reconstruction” (ILR), or the LYMPHA technique, aimed at preventing lymphedema at the time of axillary dissection.^19^^,^^14^

The analysis reveals a near-total absence of high-level evidence: no Level 1 systematic reviews or meta-analyses of randomized trials were identified, and only two studies (4%) provided Level 2 evidence. Of these, one was a pilot randomized controlled trial of robotic supermicrosurgery with 20 patients,^28^ and the other a randomized trial of immediate lymphatic reconstruction with 144 patients.^14^ The remaining 96% of articles provided Level 3–5 evidence, with case series alone accounting for 48% of all publications. This predominance of observational data is characteristic of surgical innovation, where ethical and logistical challenges, including the impossibility of blinding, the heterogeneity of disease staging, and the dependence on operator expertise, often prevent randomization.^29^ Nevertheless, the absence of Level 1 evidence underscores a critical gap: the field currently lacks the robust comparative effectiveness data necessary to establish definitive treatment algorithms. Multi-center, adequately powered randomized trials comparing LVA, VLNT, and combined approaches at defined disease stages are urgently needed to advance lymphatic surgery from a technically proficient to an evidence-based subspecialty.

**Figure 6:**
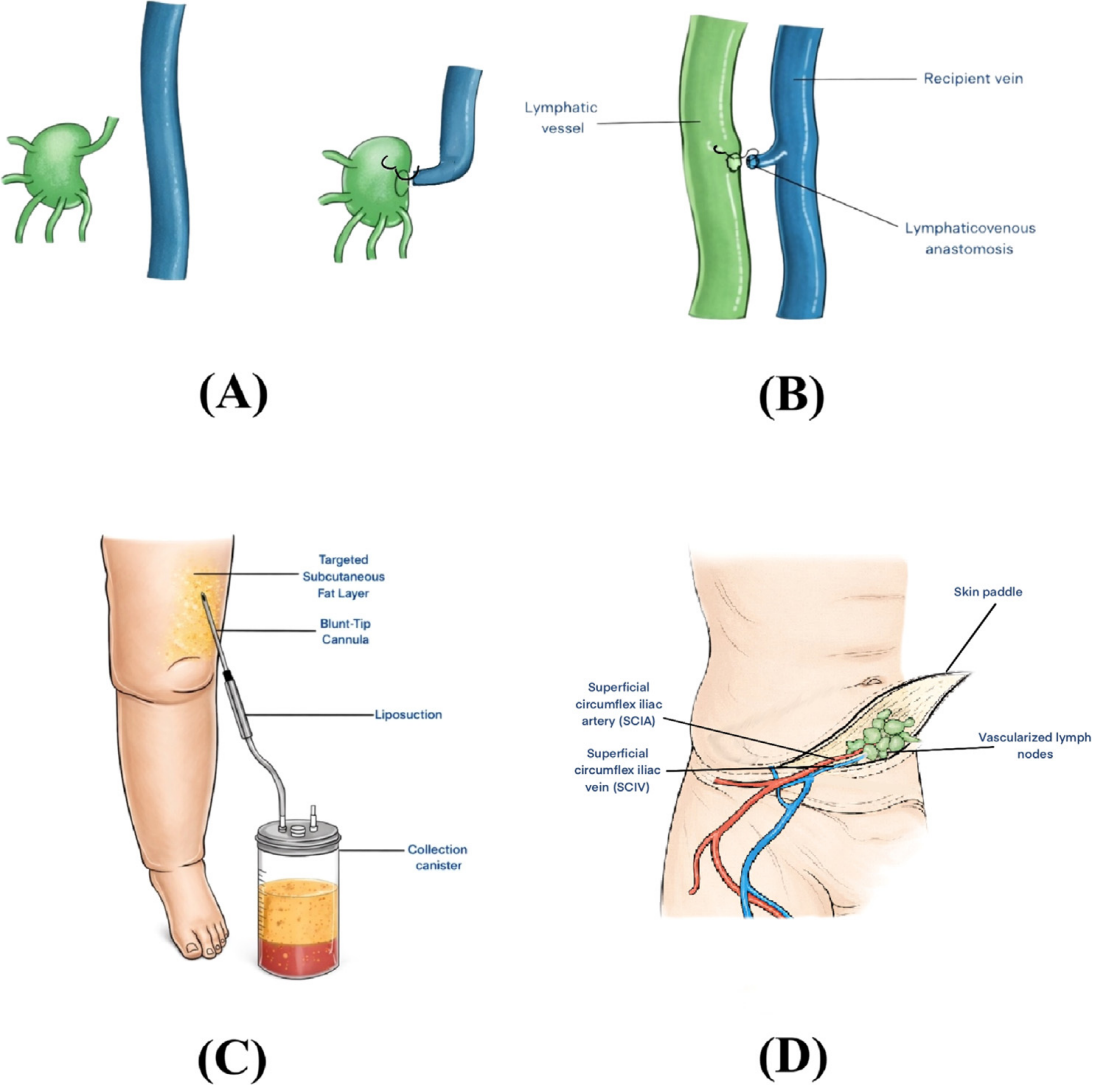
Surgical approaches for lymphedema management. (A) Lymphaticovenous anastomosis (LVA). (B) Lymph node–to–vein anastomosis (LNVA). (C) Suction-assisted lipectomy targeting subcutaneous adipose tissue. (D) Superficial circumflex iliac artery perforator (SCIP)–based vascularized lymph node transfer (VLNT) demonstrating harvest of vascularized groin lymph nodes on the SCIP pedicle.

An overview of surgical management strategies for lymphedema is shown in Figure 6. Interestingly, lymph node–to–vein anastomosis (LNVA) does not appear among the 50 most-cited publications. Rather than reflecting a lack of clinical importance, this likely reflects the relatively recent evolution of LNVA as a distinct supermicrosurgical technique; the procedure has not yet had sufficient time to accrue the citation counts necessary for inclusion in this historical analysis. Additionally, LNVA has often been reported within broader LVA series rather than as a standalone procedural entity.^27^^,^^30^

## Limitations

This study has several limitations inherent to bibliometric analyses. First, the reliance on total citation counts introduces a time bias that favors older publications, which have had a longer duration to accumulate citations. Although we have supplemented absolute counts with citation density to mitigate this bias, recently impactful advancements such as robotic supermicrosurgery may still be underrepresented. Second, the exclusive focus on the top 50 cited articles introduces an inherent selection bias; while the identified journals and themes reflect the most cited work, they may not capture the full breadth of the field’s current research activity. Third, the search was restricted to the Web of Science Core Collection, which, while considered the standard for academic metrics, does not capture all citations found in other repositories such as Scopus or Google Scholar. Fourth, the inclusion criteria were limited to English-language publications, which may have excluded valuable contributions published in other languages, particularly given the strong contribution of Asian institutions to this field. Finally, it must be noted that citation frequency reflects a study’s impact and recognition within the scientific community but does not necessarily equate to its scientific quality or current clinical validity, as demonstrated by the predominance of Level 4 and Level 5 evidence in this study.

## Conclusion

The 50 most-cited articles in lymphatic surgery chart the evolution of a specialty that has rapidly advanced from palliative debulking procedures to sophisticated, physiologic supermicrosurgery. This study highlights a distinct geographic trend, with Asian institutions, particularly in Japan and Taiwan, leading the development of foundational technical innovations such as LVA and VLNT. Keyword analysis confirms supermicrosurgery, lymphaticovenous anastomosis, and indocyanine green lymphography as the central thematic pillars of the most influential literature. The field is currently characterized by an increasing reliance on imaging-guided precision, a shifting focus toward preventive strategies such as immediate lymphatic reconstruction, and an evolution toward combined physiologic and reductive approaches. Critically, the evidence base remains grounded largely in case series and expert opinion, with only two Level 2 randomized controlled trials and no Level 1 evidence identified. Future research efforts must prioritize well-designed, multi-center prospective comparative studies to refine surgical indications and establish standardized, evidence-based treatment algorithms.

## Author contributions

Conceptualization, E.L.; methodology, A.A.Q. and P.N.; software, A.A.Q. and P.N.; validation, H.M., O.C., J.P.H. and E.L.; formal analysis, A.A.Q. and P.N.; investigation, A.A.Q.; resources, A.A.Q.; data curation, A.A.Q. and P.N.; writing and original draft preparation, A.A.Q.; writing—review and editing, A.A.Q., P.N., H.M., O.C., J.P.H. and E.L.; visualization, A.A.Q.; supervision, E.L.; project administration, E.L. All authors have read and agreed to the published version of the manuscript.

## Funding

This research received no external funding.

## Informed consent statement

Patient consent was not required as this study is a bibliometric analysis of previously published literature.

## Data availability statement

The corresponding authors can provide the data upon request.

## Declaration of competing interest

The authors declare that they have no conflicts of interest.

## Appendix Supplementary materials

Image, application 1
