# Supplementary material for: The 50 most-cited publications in lymphatic surgery: A bibliometric analysis
Source: JPRAS Open. 2026 Mar 28;49:529–49. doi: 10.1016/j.jpra.2026.03.032 (PMC13101629; doi:10.1016/j.jpra.2026.03.032)
Supplement: Supplementary file 1 [file mmc1.docx]

**Supplementary material**

**S1:**

| **Inclusion criteria** | **Exclusion criteria** |
| --- | --- |
| 1. Articles primarily focused on surgical interventions for lymphedema, including lymphaticovenous anastomosis (LVA), vascularized lymph node transfer (VLNT), lymphatic vessel transfer, suction-assisted lipectomy, and radical excision (Charles procedure). 2. Publications addressing clinical outcomes, surgical techniques, donor site anatomy, perioperative management, or safety profiles related to lymphatic surgery. Original articles, reviews, and case series directly relevant to the field. 3. Articles published in English and indexed in the Web of Science Core Collection database. | 1. Publications exclusively concerning sentinel lymph node biopsy, oncologic staging, or diagnostic mapping (e.g., lymphoscintigraphy protocols) without surgical reconstruction outcomes. 2. Studies focusing solely on conservative management (e.g., complex decongestive therapy, manual drainage) or pharmacologic therapy. 3. Editorials, letters to the editor, conference abstracts, and meeting proceedings. Purely experimental animal studies or basic science research lacking direct clinical application. |

**Search Exclusion Terms (NOT Operator)**

To ensure specificity, the following exclusion terms were applied using the NOT operator in the Web of Science search strategy:

NOT TS=(“sentinel lymph node biopsy” OR “oncologic staging” OR “manual lymph drainage” OR “complex decongestive therapy” OR “pneumatic compression”)

**S2:**


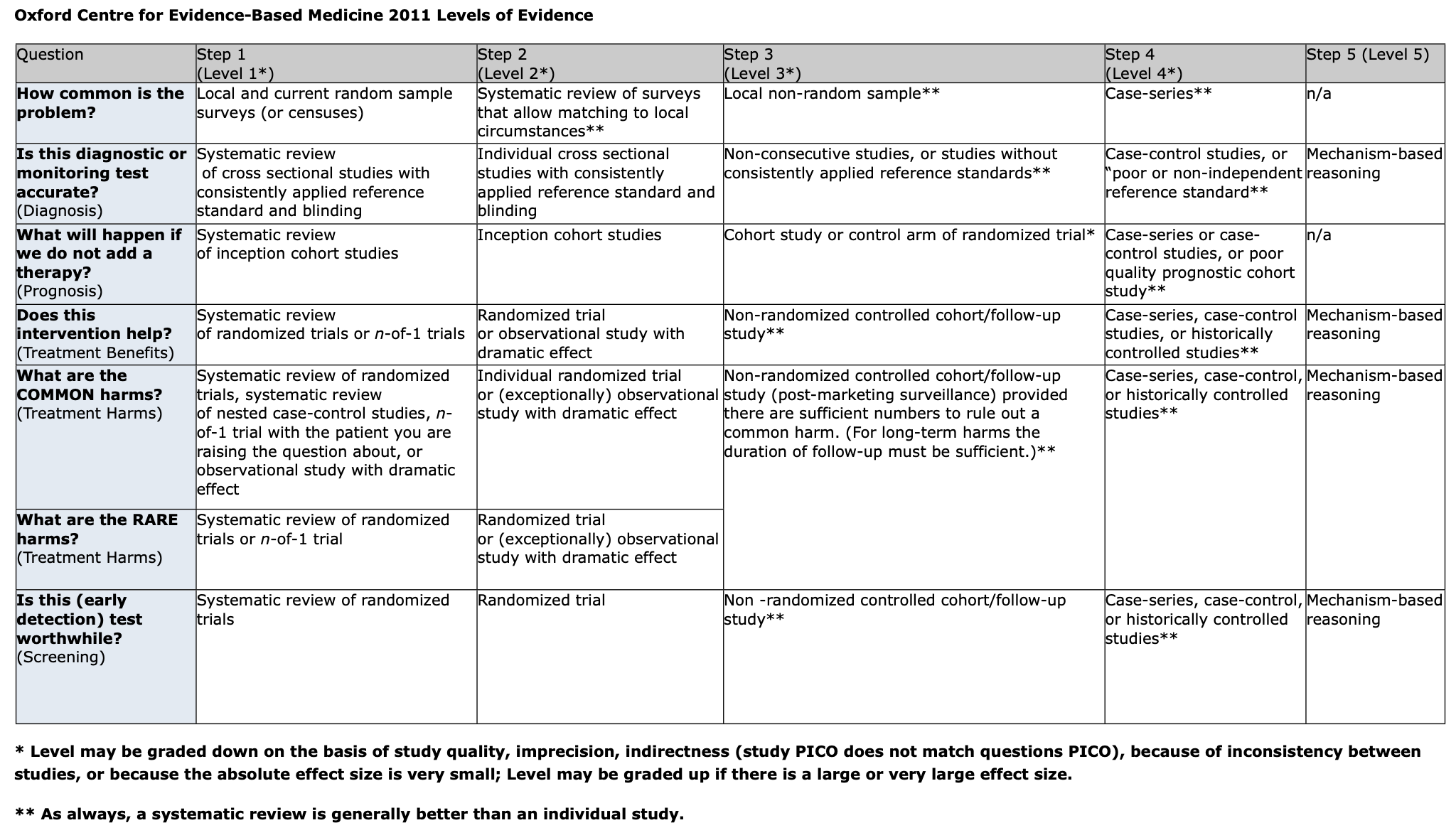


*Source: OCEBM Levels of Evidence Working Group. "The Oxford 2011 Levels of Evidence". Oxford Centre for Evidence-Based Medicine. <http://www.cebm.net/index.aspx?o=5653>. Accessed on: October 17, 2025.

**Table S1.** Overview of Included Studies: Titles and Key Findings (n=50)

| **Title** | **Summary** |
| --- | --- |
| Vascularized Groin Lymph Node Transfer Using the Wrist as a Recipient Site for Management of Postmastectomy Upper Extremity Lymphedema | This study introduces the concept of "distal" lymph node transfer. 13 patients underwent VLNT from the groin to the wrist. Results showed a statistically significant 50.55% reduction in limb circumference and a reduction in cellulitis episodes over a mean follow-up of 56 months. The authors proposed the "pump mechanism" hypothesis for lymphatic drainage. |
| Vascularized Groin Lymph Node Flap Transfer for Postmastectomy Upper Limb Lymphedema: Flap Anatomy, Recipient Sites, and Outcomes | A dual-purpose study. First, it defined the vascular anatomy of the groin lymph node flap (SCIP) using 10 cadavers. Second, it reported clinical outcomes of 10 patients receiving VLNT to the wrist or elbow, showing durable limb reduction and highlighting the importance of preserving the deep branch of the SCIA artery. |
| Perforator Flaps and Supermicrosurgery | This paper defines the field of "Supermicrosurgery" (anastomosis of vessels <0.8mm). |
| Lymphedema: Surgical and Medical Therapy | A comprehensive review by five leading experts summarizing the state of the art. It compares physiological procedures (LVA, VLNT) vs. reductive procedures (Liposuction, Charles) and establishes treatment algorithms based on the stage of lymphedema. |
| Complications of Autologous Lymph-node Transplantation for Limb Lymphoedema | A safety study. It followed 26 patients who underwent VLNT and reported a high rate of donor-site complications, specifically that some patients developed new lymphedema in the leg where the nodes were taken from. |
| Surgical Treatment of Lymphedema | Educational review describing the current "options" of surgical management. It broadly categorizes treatments into Physiologic (LVA, VLNT) vs. Debulking (Liposuction). It is widely cited because it defines the indications for each surgery (e.g., LVA for early fluid stages, Liposuction for late fibroadipose stages). |
| Microlymphatic Surgery for the Treatment of Iatrogenic Lymphedema | This paper describes the surgeon's refined techniques for harvesting nodes from the groin, thorax, and neck. It is a "how-to" paper that popularized the ALNT procedure globally. |
| Minimal invasive lymphaticovenular anastomosis under local anesthesia for leg lymphedema | A study proving LVA can be done under local anesthesia. They treated 52 patients and showed it was effective (82.5% improvement) even for Stage III/IV patients, challenging the idea that LVA only works for early stages. |
| Minimally Invasive Lymphatic Supermicrosurgery (MILS): Indocyanine Green Lymphography-Guided Simultaneous Multisite Lymphaticovenular Anastomoses via Millimeter Skin Incisions | Introduced "Minimally Invasive Lymphatic Supermicrosurgery" (MILS) using ICG lymphography to guide 2-mm skin incisions for multiple lymphaticovenular anastomoses (LVA). The technique allows for simultaneous multisite LVAs (average 3.6 per limb) with minimal invasiveness and good short-term patency. |
| First-in-human robotic supermicrosurgery using a dedicated microsurgical robot for treating breast cancer-related lymphedema: a randomized pilot trial | The first Randomized Controlled Trial (RCT) comparing Robot-Assisted LVA vs. Manual LVA. It showed the robot was feasible and improved the speed of anastomosis over time. |
| A case of donor-site lymphoedema after lymph node superficial circumflex iliac artery perforator flap transfer | It reports a confirmed case of donor-site lymphedema in the leg after harvesting a groin flap (SCIP) for VLNT. This paper was crucial in pushing surgeons to adopt "Reverse Lymphatic Mapping" to avoid damaging the leg's drainage during harvest. |
| Donor-Site Lymphatic Function after Microvascular Lymph Node Transfer | Investigated the safety of taking lymph nodes from the groin. They followed 13 patients who donated nodes and used lymphoscintigraphy to check their legs. They found that while no clinical lymphedema occurred, there was measurable slowing of lymph flow in the donor legs, highlighting the sub-clinical risk of the procedure. |
| Vascularized Lymph Node Transfer Based on the Hilar Perforators Improves the Outcome in Upper Limb Lymphedema | A technical refinement study. It compared 11 patients who had standard groin flaps vs. 10 patients who had hilar perforator-based flaps (specifically targeting the tiny vessels feeding the nodes). The hilar group had significantly better outcomes (circumference reduction), proving that precise blood supply to the nodes matters. |
| The Intravascular Stenting Method for Treatment of Extremity Lymphedema with Multiconfiguration Lymphaticovenous Anastomoses | Introduced the "Intravascular Stenting" technique for LVA. This involves inserting a tiny nylon thread (stent) into the lymphatic vessel to keep it open while suturing, making it easier to perform side-to-end or end-to-end anastomoses. It allows for "multiconfiguration" (complex) hookups to drain flow from both directions. |
| Barcelona Consensus on Supermicrosurgery | Defined supermicrosurgery as a technique for vessels of 0.3 to 0.8 mm and single nerve fascicles. Confirmed applications for lymphedema, nerve reconstruction, and distal replantation. |
| Multisite Lymphaticovenular Bypass Using Supermicrosurgery Technique for Lymphedema Management in Lower Lymphedema Cases | A large series of 84 patients (lower limb). It advocated for "Multisite" LVA, meaning performing anastomoses at multiple levels of the leg (groin, knee, ankle) simultaneously. They showed that increasing the number of bypasses correlates with better volume reduction. |
| Current Concepts in the Surgical Management of Lymphedema | Educational review standardizing the treatment algorithm (LVA vs VLNT vs Liposuction) for plastic surgeons. |
| Microsurgery for treatment of peripheral lymphedema: Long-term outcome and future perspectives | A thorough report covering 30 years of experience and over 1,500 patients. Corradino Campisi is a European pioneer. This paper describes his specific technique of "Lymphatic-Venous-Lymphatic-Plasty" and reports long-term stability in outcomes. It is historically significant as it bridges the gap between the 1970s techniques and modern supermicrosurgery. |
| Lymphaticovenous anastomosis to prevent cellulitis associated with lymphoedema | Lymphaticovenous anastomosis (LVA) significantly reduced the frequency of cellulitis episodes in lymphedema patients (mean episodes decreased from 1.46 to 0.18 per year; P < 0.001). |
| Comparison of Vascularized Supraclavicular Lymph Node Transfer and Lymphaticovenular Anastomosis | Direct comparison showing VLNT was superior to LVA for advanced (Stage III) lower limb lymphedema. |
| Quality of life following liposuction and conservative treatment of arm lymphedema | Liposuction combined with controlled compression therapy (CCT) achieved complete reduction of arm lymphedema, significantly superior to CCT alone (50% reduction). Surgery significantly improved quality of life, particularly in physical qualities related to volume reduction and daily activities. |
| From Theory to Evidence: Long-Term Evaluation of the Mechanism of Action and Flap Integration of Distal Vascularized Lymph Node Transfers | Used ICG to prove the "Pump Mechanism" of distal VLNT in 20 patients. |
| Simultaneous multi-site lymphaticovenular anastomoses for primary lower extremity and genital lymphoedema complicated with severe lymphorrhea | Simultaneous multi-site lymphaticovenular anastomosis (LVA) under local anesthesia effectively improved symptoms of edema and severe lymphorrhea in patients with primary lower extremity and genital lymphedema. |
| Modified Charles procedure and lymph node flap transfer for advanced lower extremity lymphedema | A modified Charles procedure (preserving superficial veins) combined with vascularized lymph node transfer was performed on 24 patients with advanced lymphedema. The method achieved maximum reduction of lymphedema with no recurrence at 14 months follow-up and no major complications. |
| A modified side-to-end lymphaticovenular anastomosis | A modified side-to-end (S-E) anastomosis technique was performed on 14 limbs. The technique allows for easier diversion of lymph flow into the venous circulation without requiring supermicrosurgical anastomosis (11-0 or 12-0 sutures). The Lower Extremity Lymphedema (LEL) index significantly decreased postoperatively (P < 0.001). |
| Overview of Lymph Node Transfer for Lymphedema Treatment | Reviews the literature on vascularized lymph node transfer (VLNT). Concludes that VLNT is a promising physiologic treatment for lymphedema, particularly when lymphovenous anastomosis (LVA) is limited by fibrosis in advanced stages. Discusses mechanisms of action including lymphangiogenesis and the "pump" theory. |
| Lymph Flow Restoration after Tissue Replantation and Transfer: Importance of Lymph Axiality and Possibility of Lymph Flow Reconstruction without Lymph Node Transfer or Lymphatic Anastomosis | Lymph flow can be spontaneously restored after tissue replantation or free flap transfer without specific lymphatic anastomosis. "Compatible lymph axiality" (aligning the flap's lymphatics with the recipient site's lymphatics) was identified as a key factor for this restoration (100% accuracy in prediction). |
| Outcomes of Lymphedema Microsurgery for Breast Cancer-related Lymphedema With or Without Microvascular Breast Reconstruction | Microsurgical breast reconstruction did not significantly improve lymphedema outcomes compared to lymphedema surgery alone. Vascularized lymph node transfer (VLNT) provided greater improvements in limb volume and cellulitis reduction compared to lymphovenous anastomosis (LVA) or conservative therapy. |
| A prospective study on combined lymphedema surgery: Gastroepiploic vascularized lymph nodes transfer and lymphaticovenous anastomosis followed by suction lipectomy | A prospective non-randomized comparative study of 37 patients (21 combined group, 16 control) evaluating a combined surgical approach of gastroepiploic vascularized lymph node transfer (VLNT) and lymphaticovenous anastomosis (LVA) followed by suction lipectomy for upper and lower extremity lymphedema. The combined physiologic-reductive strategy demonstrated significant volume reduction and improved functional outcomes compared to physiologic procedures alone. |
| NEAR-INFRARED ILLUMINATION SYSTEM-INTEGRATED MICROSCOPE FOR SUPERMICROSURGICAL LYMPHATICOVENULAR ANASTOMOSIS | The use of an operating microscope with an integrated near-infrared illumination system significantly reduced the time required to detect and dissect lymphatic vessels (2.3 min vs. 6.5 min; P=0.010) compared to conventional methods, although the final lymphedema reduction outcomes were similar between groups. |
| Circumferential suction-assisted lipectomy for lymphoedema after surgery for breast cancer | Circumferential suction-assisted lipectomy (liposuction) combined with lifelong compression therapy is highly effective for end-stage, non-pitting breast cancer-related lymphedema. The mean reduction in excess arm volume was 118% at 12 months, with no recurrence of swelling. |
| Evaluating the Impact of Immediate Lymphatic Reconstruction for the Surgical Prevention of Lymphedema | Immediate lymphatic reconstruction (ILR) performed at the time of axillary lymph node dissection resulted in a lymphedema rate of 3.1% in a high-risk population of 97 women, suggesting it is a promising preventive approach. |
| Changing the Paradigm: Lymphovenous Anastomosis in Advanced Stage Lower Extremity Lymphedema | Functioning lymphatic vessels were successfully identified in advanced-stage lymphedema using ultrasound and MR lymphangiography. Lymphovenous anastomosis (LVA) resulted in a significant volume reduction (15.5% at 1 year) and a decrease in cellulitis frequency (0.84 to 0.07/year), suggesting LVA is effective even for advanced stages. |
| Complete lymph flow reconstruction: A free vascularized lymph node true perforator flap transfer with efferent lymphaticolymphatic anastomosis | Reported the first case of complete lymph flow reconstruction using a true perforator lymph node flap (from lateral thorax) with efferent lymphaticolymphatic anastomosis (ELLA) to the recipient's iliac lymphatic vessel. The procedure successfully reduced lymphedema volume (LEL index 306 to 264) and prevented cellulitis recurrence. |
| Controlled compression and liposuction treatment for lower extremity lymphedema | Liposuction combined with controlled compression therapy (CCT) is highly effective for reducing lower extremity lymphedema, achieving complete reduction of excess volume (100%) in contrast to CCT alone. The reduction was maintained long-term without recurrence. |
| Lymphedema | Provides a comprehensive overview of lymphedema pathophysiology, diagnosis, and management, emphasizing the need for a multimodal approach and highlighting emerging therapies. |
| Efficacy of Immediate Lymphatic Reconstruction to Decrease Incidence of Breast Cancer-related Lymphedema | Investigated immediate lymphatic reconstruction (ILR) at the time of axillary lymph node dissection (ALND). Found that ILR significantly decreased the incidence of breast cancer-related lymphedema (BCRL) compared to a control group (e.g., 9% vs 32% in some cohorts, though exact numbers vary by specific publication year/update). |
| Navigation lymphatic supermicrosurgery for iatrogenic lymphorrhea: Supermicrosurgical lymphaticolymphatic anastomosis and lymphaticovenular anastomosis under indocyanine green lymphography navigation | Intraoperative indocyanine green (ICG) lymphography was used to identify ruptured lymphatic vessels causing intractable inguinal lymphorrhea in 4 patients. Supermicrosurgical anastomosis (LVA or lymphaticolymphatic) successfully cured lymphorrhea in all cases with a significantly shorter treatment duration (5.0 days) compared to conservative treatment (30.0 days). |
| Free lymph node flap transfer and laser-assisted liposuction: a combined technique for the treatment of moderate upper limb lymphedema | Described a combined approach of VLNT and laser-assisted liposuction for moderate upper limb lymphedema. The technique was safe and effective, offering both volume reduction (from liposuction) and physiologic improvement (from VLNT), potentially reducing the need for conservative therapy. |
| Indication of Lymphaticovenous Anastomosis for Lower Limb Primary Lymphedema | Investigated LVA efficacy in primary lower limb lymphedema. Found that LVA was effective in patients with onset age >11 years and those with leg dermal backflow stage 2 or "no backflow" pattern on ICG lymphography. Patients with onset <11 years or "distal backflow" pattern had poorer outcomes. |
| Indocyanine Green Lymphographic Evidence of Surgical Efficacy Following Microsurgical and Supermicrosurgical Lymphedema Reconstructions | Used ICG lymphography to evaluate surgical efficacy. Demonstrated that effective LVA or VLNT results in observable improvements in lymphatic drainage patterns (e.g., appearance of linear channels, reduction of dermal backflow) on postoperative ICG lymphography, providing objective evidence of physiologic improvement. |
| Efferent Lymphatic Vessel Anastomosis Supermicrosurgical Efferent Lymphatic Vessel-to-Venous Anastomosis for the Prophylactic Treatment of Subclinical Lymphedema | Performed efferent lymphatic vessel anastomosis (ELVA) on 14 legs with subclinical lymphedema. The procedure was successful in all cases, and all legs remained free from symptomatic lymphedema at 1-year follow-up, suggesting ELVA is effective for prophylaxis. |
| Successful treatment of early-stage lower extremity lymphedema with side-to-end lymphovenous anastomosis with indocyanine green lymphography assisted | Intraoperative indocyanine green (ICG) lymphography was used to confirm patency of side-to-end lymphovenous anastomoses (LVA) in 5 patients with early-stage lower extremity lymphedema. The mean reduction rate was 63.8% at 10 months follow-up, with no cellulitis episodes observed. |
| Comparison of Outcomes between Side-to-End and End-to-End Lymphovenous Anastomoses for Early-Grade Extremity Lymphedema | Compared side-to-end (STE) and end-to-end (ETE) LVA configurations. Found that STE LVA resulted in significantly greater improvement in limb circumference reduction (3.2% vs 2.2%) compared to ETE in early-grade lymphedema, likely due to bidirectional flow. |
| The lymphatic superficial circumflex iliac vessels deep branch perforator flap: A new preventive approach to lower limb lymphedema after groin dissection-preliminary evidence | Described a modified SCIP flap (L-SCIP) containing lymphatic vessels for immediate reconstruction after groin dissection for vulvar cancer. Preliminary results suggested a significant protective effect against secondary lower limb lymphedema compared to the contralateral side or historical controls. |
| Double gastroepiploic vascularized lymph node tranfers to middle and distal limb for the treatment of lymphedema | Compared single vs. double gastroepiploic vascularized lymph node transfer (VLNT). The double transfer technique (inset at middle and distal limb) resulted in significantly greater limb circumference reduction (43.76%) compared to single transfer, with no increase in donor site morbidity. |
| Risk of donor-site lymphatic vessel dysfunction after microvascular lymph node transfer | Investigated donor site morbidity after groin lymph node harvest. Found that even with a modified, more conservative harvest technique, subclinical signs of lymphatic dysfunction (abnormal lymphoscintigraphy) could be observed in the donor limb, highlighting the importance of minimizing surgical exploration range. |
| Surgical management of lymphedema | A narrative review providing a comprehensive overview of the surgical management of lymphedema, discussing the pathophysiology, staging, and various treatment options including microsurgical (LVA, VLNT) and excisional (liposuction, Charles procedure) approaches, with emphasis on patient selection and multidisciplinary management. |
| Navigation Lymphatic Supermicrosurgery for the Treatment of Cancer-Related Peripheral Lymphedema | A case series of 8 patients (21 LVAs) using indocyanine green (ICG) lymphography-guided supermicrosurgical lymphaticovenular anastomosis for cancer-related peripheral lymphedema. ICG navigation enabled real-time identification of functional lymphatic vessels, facilitating precise surgical planning and effective volume reduction. |
| The “Octopus” Lymphaticovenular Anastomosis: Evolving Beyond the Standard Supermicrosurgical Technique | A pilot case series of 9 patients describing the “Octopus” LVA technique, which connects multiple lymphatic channels to a single vein simultaneously. This multi-input configuration maximizes lymphatic outflow through a single anastomotic site, representing a technical evolution beyond standard end-to-end or side-to-end supermicrosurgical approaches. |
